# Fungi Occurrence in Ready-to-Eat Hazelnuts (*Corylus avellana*) From Different Boreal Hemisphere Areas

**DOI:** 10.3389/fmicb.2022.900876

**Published:** 2022-04-26

**Authors:** Silvia Jane Lombardi, Gianfranco Pannella, Patrizio Tremonte, Ida Mercurio, Franca Vergalito, Costantino Caturano, Lucia Maiuro, Massimo Iorizzo, Mariantonietta Succi, Elena Sorrentino, Raffaele Coppola

**Affiliations:** Department of Agricultural, Environmental and Food Sciences, University of Molise, Campobasso, Italy

**Keywords:** *Aspergillus*, *Penicillium*, *Fusarium*, *Alternaria*, *Rhizopus*, *Trichoderma*, nuts

## Abstract

The present study evaluated the fungal contamination of ready-to-eat dried hazelnuts considering for the first time the application of the same condition drying process of several hazelnut cultivars from different boreal hemisphere areas. Fifty lots of hazelnuts (*Corylus avellana*), belonging to eight cultivars from seven regions in four countries, were analyzed for fungal microbiota, describing both load levels and species diversity. For this purpose, a polyphasic approach consisting of morphological examination (optical and scanning electron microscope observation) and molecular characterization [PCR-DGGE analysis and sequence analyses of the internal transcribed spacer (ITS)] was performed. The results show that different fungal populations occur in dried hazelnuts regardless of their geographical area of production. Although some varieties appear to be relatively less susceptible, species related to *Aspergillus*, such as *A. commune* and *A. ochraceus*, *Penicillium*, including *P. commune, P. solitum*, and *P. expansum*, and *Rhizopus*, for instance, *R. stolonifer* and *R. oryzae*, have generally been found. A related character “hazelnut cultivar—fungi” was found for species related to the genera *Trichoderma* and *Fusarium*, including *F. oxyxporum*, *F. solani*, and *F. falciforme*. All 14 species found are known to host pathogenic strains. Therefore, their presence in a ready-to-eat product, such as dried hazelnuts, can pose a real danger to the consumer. Based on these considerations, the development of new protective strategies seems highly desirable. The species-level description of the contaminating fungal community acquired through this study is the starting point for the development of tailor-made protective biotechnologies.

## Introduction

The European hazelnut (*Corylus avellana*) is a crop of major interest in the agri-food sector in several countries (Food and Agriculture Organization of the United Nations [FAO], 2020). The main hazelnut-producing countries are in the northern hemisphere (Turkey, Italy, the United States, Georgia, and Spain). Noteworthy in the southern hemisphere is the hazelnut production in Chile.

Based on historical and forecast data, the demand for hazelnuts is steadily growing. A large part of the production is used in the confectionery industry (chocolate, spreads, pralines, etc.). In addition, the demand for hazelnuts, offered as a ready-to-eat product, is increasing significantly. The growing interest in whole hazelnuts is due to the nutritional and nutraceutical properties of the nuts (unsaturated fatty acids, polyphenols, phytosterols, vegetable fiber, and micronutrients). Hazelnuts contain all these nutrients, and their consumption might help produce several beneficial effects such as immunologic and inflammatory response regulation, blood cholesterol reduction, and incidence of cardiovascular disease (Phillips et al., 2005; Lelli et al., 2021). Ready-to-eat hazelnuts can be proposed raw or can be processed by thermal treatments, such as roasting or drying and others.

Several authors reported that the roasting process significantly affects peroxide value, free fatty acid, thiamine, riboflavin, polyphenolic contents, and total amino composition of hazelnuts, as well as at a certain temperature could lead to acrylamide formation (Özdemir et al., 2001; Pelvan et al., 2012; Tepe et al., 2020). In addition, although roasting eliminates microorganisms, it is not able to degrade heat-resistant mycotoxins (Siciliano et al., 2017). Aflatoxin levels exceeding the legal threshold have often been found in nuts (Abdulkadar et al., 2000). Based on this consideration, it is crucial to reduce humidity content, water activity, and consequently microbial load within 72 h after harvesting. Commonly, dehulled and raw hazelnuts are dried until 6% moisture content. Moreover, drying treatment assures physical–chemical conditions (moisture content and water activity) for retaining bioactive compounds and minimizing lipid oxidation and enzyme activity (Wang et al., 2018). Therefore, this treatment is considered essential to ensure the hazelnuts’ quality (Turan, 2018; Chen et al., 2021). As reported earlier, the effect of drying on enzymatic, chemical, and physical–chemical conditions is well-recognized and a temperature of 40–45°C at 40% RH could be considered as the recommended process conditions (Mousadoost et al., 2018; Wang et al., 2018; Valente et al., 2020). The drying temperature (usually under 50°C) is appreciated by the modern consumer, who prefer minimally processed foods, in which heat treatment is minimized in favor of the organoleptic properties and nutrients content of the product. Knowledge about the effects of the drying process on microbiological safety and the avoidance of mycotoxins is still limited. A specific challenge test has been applied to evaluate the drying effect on certain mycotoxigenic molds (Valente et al., 2020). However, the hazelnut microbiota depends on several factors related to the cultivar, the geographical area, and pre- and post-harvest practices. A wide range of fungi, such as *Aspergillus*, *Penicillium*, *Rhizopus*, *Ulocladium*, *Alternaria*, *Drechselera*, *Trichothecium*, *Scopulariopsis*, *Cladosporium*, and *Mucor*, were revealed (Botondi, 2019; Wiman et al., 2019; Saffari et al., 2021). In addition, the heat resistance of microorganisms should be considered; heat resistance generally produces heat-resistant spores that can be activated by thermal treatment allowing them to germinate and spoil the products (Rico-Munoz and dos Santos, 2019). The information available to date on the sanitation effect of drying processes is often not comparable due to the significantly different variables adopted in each study. Based on these assumptions, the aim of this article was to assess the fungal contamination of hazelnuts subjected to the same drying treatment and harvested in different geographical areas.

## Materials and Methods

### Sampling

**FIGURE 1:**
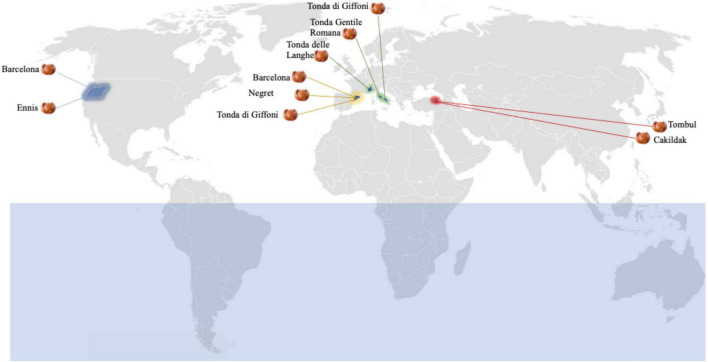
Dried hazelnut lots from different boreal hemisphere areas: 10 Barcelona lots from Catalunya, Spain (five) and Oregon, United States (five); five Cakildak lots from Ordu, Turkey; five Ennis lots from Oregon, United States; five Negret lots from Catalunya, Spain; five Tombul lots from Giresum, Turkey; five Tonde delle Langhe lots from North Italy; five Tonda Gentile Romana lots from Central Italy; 10 Tonda di Giffoni lots from Southern Italy (five) and Catalunya, Spain (five).

Fifty lots of hazelnuts (*C. avellana*) referable to eight cultivars (Barcelona, Çakildak, Ennis, Negret, Tombul, Tonda delle Langhe, Tonda Gentile Romana, and Tonda di Giffoni), from seven regions of four countries (Italy, Spain, Turkey, and the USA) were purchased from 10 different processing companies (Figure 1). Five lots of hazelnut kernels were taken from each plant that was selected based on their drying process. In all cases, unshelled hazelnuts have been obtained from the same drying process using forcing ventilation of hot air at 40°C to obtain a kernel moisture content of about 6.0%.

For each sample (n. 5) from each processing company (n. 10), moisture content was determined using an oven at 105°C (Official Methods of Analysis, 2005). Water activity (a_*w*_) values were determined using AquaLab CX-2 (Decagon Devices, Pullman, WA, United States) at a constant temperature of 25°C.

The hazelnuts, having an average diameter of about 1.00 ± 0.10 cm and an average weight of 0.50 ± 0.10 g, were packed in polyethylene bags and stored at room temperature.

### Fungi Enumeration

An assessment of the level of fungi contamination was carried out by suspending 25 g from each hazelnut sample in 225 ml of sterile physiological water (NaCl, 0.9 g/L) used as a dilution solution. Homogenized samples were diluted and inoculated on Dichloran Rose Bengal Agar (DRBA- Oxoid, Milan, Italy) by the pour plate method for enumeration. The plates were inoculated and incubated at 25°C for 5–7 days. For each sample, the analyses were carried out in triplicate. Thereafter, all purified cultures were retained at 4°C on MEA agar plates (Malt extract agar; Oxoid, Milan, Italy). Fungi counts from plates, between 15 and 150 colony-forming units (CFU), were used in calculating the total fungi count per g in each sample.

### Fungi Isolation and Identification

Representative numbers (10%) of colonies, randomly picked from each plate having between 15 and 150 colony-forming units (CFU) medium, were purified by streaking on the DRBA media. The colonies were randomly selected according to morphological differences (shape, size, color of the colony, and shape of cells).

A polyphasic approach consisting of morphological examination and molecular typing was adopted for elucidating the diversity of fungi in the hazelnut samples.

#### Morphological Examination

For taxonomic purposes, the isolated fungi were subjected to observation under an optical microscope (Olympus, with interferential contrast) to analyze the morphological characteristics specific to the fungal genus. The dichotomous identification key for imperfect fungi (Illustrated Genera of Imperfect Fungi) was used to identify the isolates. All fungal isolates were cultivated on MEA, assessed for macroscopic and microscopic characters, and compared with descriptions in appropriate keys (Samson et al., 2011, 2019). The characteristics of the hyphae, the fruiting organs, the shape of the conidia, and spores were observed by an optical microscope and by a scanning electron microscope. For this purpose, culture samples of isolates were harvested from the culture medium by centrifugation, washed in sterile non-ionic water two or three times, and fixed in 3% glutaraldehyde(v/v) in 0.1 M sodium phosphate buffer for 12 h. The samples were then rinsed three times with the same buffer and dehydrated (two times for each solution, 10 min each) in a graded ethanol series (40, 60, 80, 90, 95, and 100%) with the last wash in acetone for a better CO_2_ substitution during the drying process at a pressure of 1,200 bars. After drying in a CO_2_ critical point (Emitech K850), samples were sputter coated with palladium gold in Emitech K550 and observed in a scanning electron microscope (SEM Zeiss DSM 940A) operating at 10 kV.

#### Genotypic Characterization

Biodiversity in species among all the isolates was ascertained through multiple culture-dependent approaches, consisting of polymerase chain reaction-denaturing gradient gel electrophoresis (PCR-DGGE) and internal transcribed spacer (ITS) region gene sequencing.

##### DNA Extraction From Pure Fungal Cultures

The DNA from pure fungal cultures was extracted using a Fungi/Yeast Genomic DNA Isolation Kit (Norgen Biotek, Ontario, Canada) following the procedure of Kumar and Mugunthan (2018). In brief, 5 ml of collection solution was poured on the culture plate and the fungal mycelium was centrifugated at 14,000 rpm for 1 min and the pellet, containing the fungal mycelium, was recovered and then resuspended in 250 μl of resuspension solution A. One hundred fifty microliters of proteinase K and 200 units of lyticase enzyme (Sigma Aldrich, St. Louis, Mo, United States) were added to the mixture and incubated for 45 min at 37°C. Then, to this mixture, 500 μl of lysis buffer and glass beads were added, and it was vortexed for 10 min at maximum speed followed by 2 h of incubation at 90°C with intermittent vortexing.

The tubes, containing the mixture, were placed in an ultrasonicator water bath (Bionics) and sonicated for 20 min. The mixture was centrifugation for 2 min at 14,000 rpm and the supernatant was transferred to a fresh microcentrifuge tube with an equal volume of ethanol and mixed. The lysate was placed in the spin column and centrifuged at 10,000 rpm for 1 min followed by two washes with 500 μl of wash buffer. The DNA was recovered in 100 μl of elution buffer by spinning at 10,000 rpm for 2 min and was stored at -20°C prior to the subsequent analyses.

##### Polymerase Chain Reaction-Denaturing Gradient Gel Electrophoresis Analysis

A fragment of a region of the 28S rDNA gene (260 bp fragment) was amplified using a couple of eukaryotic universal primers: the forward primer U1 (5′-CGC CCG CCG CGC GCG GCG GGC GGG GCG GGG GTG AAA TTG TTG AAA GGG AA-3′) and the reverse primer U2 (5′-GAC TCC TTG GTC CGT GTT-3′) (Sandhu et al., 1995; Wu et al., 2002; Li et al., 2008). A 30-bp GC clamp was attached to the 5′ end of the U1 primer (Sheffield et al., 1989). The PCR reaction was performed in a final volume of 50 μl containing 2.5 μl of DMSO, 0.4 mM of each primer, 200 mM of the deoxyribonucleotide triphosphate, 3 mM of MgCl_2_, 5 ml of 10× of reaction Taq buffer MgCl_2_ free (Promega), 1.25 U of Taq DNA polymerase (Promega), and 2 μl of the extracted DNA. PCR was conducted in a Mastercycle (Eppendorf, Hamburg, Germany) with the following program: initial denaturation at 94°C for 3 min, followed by 30 cycles of denaturation at 94°C for 45 s, annealing at 50°C for 50 s, and extension at 72°C for 90 s. PCR program ended with the final extension at 72°C for 5 min (El Sheikha et al., 2011). The PCR amplicons were separated by denaturing gradient gel electrophoresis (DGGE) using the Biorad DCodeTM Universal Mutation Detection System (BioRad, Hercules, CA, United States) as described by Iorizzo et al. (2020a). Put briefly, electrophoresis was performed in a 0.8-mm polyacrylamide gel (8% w/v acrylamide-bisacrylamide 37.5:1) by using two different ranges of denaturant to optimize the separation of the products. Two denaturant gradients, from 40 to 60% (100% denaturant was 7 M urea plus 40% w/v formamide) increasing in the direction of electrophoresis, were used. The gels were subjected to a constant voltage of 120 V for 5 h at 60°C, and after electrophoresis, they were stained for 20 min in 1.25 × TAE containing 50 μg/ml ethidium bromide and visualized under UV illumination. DGGE gels were digitalized by GEL DOC XR System using the software Quantity One Analysis Version 4.6.7 (Bio-Rad Laboratories, Inc.) and analyzed with the pattern analysis software package, Gel Compare II Version 2.0 (Applied Maths, Kortrijk, Belgium). The calculation of similarities in the profiles of bands was based on a Pearson product-moment correlation coefficient. Dendrograms were obtained by mean of the Unweighted Pair Group Method using the Arithmetic Average (UPGMA) clustering algorithm.

##### DNA Sequencing of the Internal Transcribed Spacer

The representative isolates from clusters obtained by DGGE analysis were submitted to PCR and sequence analyses of the internal transcribed spacer (ITS) region ITS1—5.8S—ITS2 rDNA. The ITS regions of approximately 600 bp were amplified with primers ITS4 (5′-TCCTCCGCTTATTGATATGC-3′) and ITS5 (5′-GGAAGTAAAAGTCGTAACAAGG-3′) (White et al., 1990). The amplifications were performed in 10× buffer, 15 mM MgCl2, 0.2 mM of each dNTP, 10 picomolar of ITS4 and ITS5 primer, 2 U Taq DNA Polymerase, and 2 μl of extracted DNA. The PCR reaction was carried out using a Mastercycle (Eppendorf, Hamburg, Germany) with conditions as follows: initiation for 10 min at 94°C, 35 cycles of denaturation for 1 min at 94°C, annealing for 30 s at 55°C, and extension for 1 min at 72°C, followed by a final extension step at 72°C for 10 min. The PCR products were purified using the QIAquick PCR purification kit (QIAGEN GmbH, Hilden, Germany) and were sent to a commercial facility for sequencing (Eurofins MWG Biotech Company, Ebersberg, Germany).

##### Phylogenetic Analysis

The ITS region of 17 isolates (*Penicillium* spp., *N* = 4; *Aspergillus* spp., *N* = 4; *Rhizopus* spp., *N* = 2; *Trichoderma* spp., *N* = 1; *Fusarium* spp., *N* = 3; *Alternaria* spp., *N* = 3) was used in the phylogenetic analysis. For this purpose, a multiple sequence alignment was constructed using the ClustalW algorithm, with a gap open penalty of 15 and a gap extension penalty of 6.66. Phylogenetic tree calculation was performed with the Neighbor-Joining method (Saitou and Nei, 1987) using MEGAX software (Stecher et al., 2020). The statistical significance of the phylogenetic tree was tested by using bootstrap analysis (Felsenstein, 1985), with each bootstrap value reflecting the confidence of each branch.

### Statistical Analyses

Data obtained from three independent experiments were analyzed using the software RStudio (v 3.6.3) (R Core Team, 2020). Analysis of variance (ANOVA) followed by the Tukey HDS *post-hoc* test was used to evaluate differences between samples. The significance level for all statistical tests was set to an α of 0.05. The packages ggplot2 (Wickham, 2009) and ggalluvial (Brunson and Read, 2020) were used for the graphical representation of data (Pannella et al., 2020).

## Results and Discussion

### Dried Hazelnuts and Fungi Contamination

**FIGURE 2:**
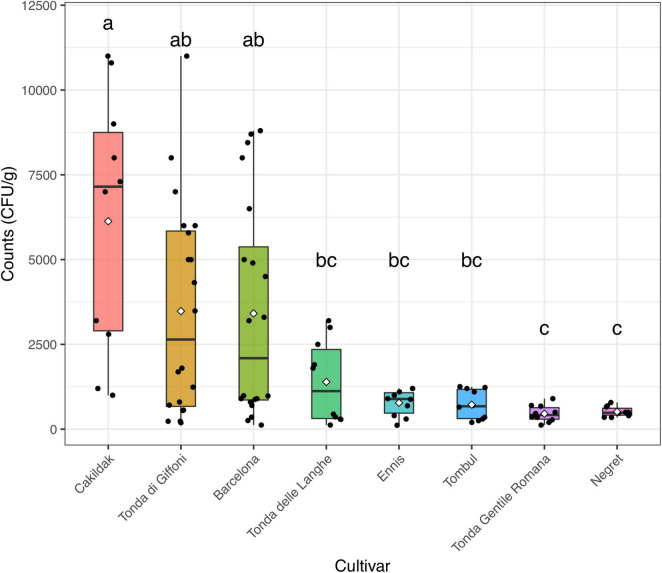
Box and jitter plots representing fungi occurrence (CFU/g) in hazelnuts samples from different cultivars. In a boxplot, the bottom and top of the boxes represent the quartiles (25th and 75th percentile), with the line and rhombus inside the box representing the median and mean, respectively. Different letters show significant differences determined by the ANOVA test (*P* < 0.05) followed by the Tukey HDS *post-hoc* test.

The moisture content and the a_*w*_ detected in the different lots of hazelnuts were calculated as the mean values of each sample (n. 5) from each processing company (n. 10). The moisture and a_*w*_ mean values, of about 6% and 0.82, respectively, resulted in an agreement with those commonly found in other dried hazelnuts (European Commission, 2002; Codex Alimentarius Commission [CAC], 2005). Regardless of both origin and cultivar, the results highlighted that the drying process guarantees a_*w*_ values in line with those recommended for ready-to-eat hazelnuts. These data are consistent with those reported by other authors (Turan, 2018; Wang et al., 2018), who believe that drying hazelnuts represent the most common technique to remove fungal contamination and would have major effects on the reduction of health-related risks. However, in our study, fungi enumeration highlighted that the contaminations remained after the drying process in samples from all assayed batches. Figure 2, which reported fungi occurrence (CFU/g) in dried hazelnut samples from different cultivars, points out that the count levels range from 0.5^2^ to 1.0^4^ CFU/g. Although fungi contaminations were found everywhere, some differences depending on diverse cultivars were appreciated. A high data dispersion was found for fungi levels detected in Cakildak, Tonda di Giffoni, and Barcelona, which showed a large interquartile range. The samples from Cakildak were characterized by the highest values showing mean and median values of about 6.1^3^ and 7.2^3^ CFU/g, respectively. The largest interquartile range was detected for the samples from “Tonda di Giffoni,” which highlighted count levels between 0.5^2^ and 1.0^4^ CFU/g with mean and median values of about 3.5^3^ and 2.6^3^ CFU/g, respectively. On the other hand, the smallest interquartile range was detected for the samples from cultivars Ennis, Tombul, Negret, and Tonda Gentile Romana. Moreover, it should be noted that the samples referable to these last four cultivars are also characterized by the lowest levels of contamination. A relationship between cultivars and mold presence has already been pointed out and/or hypothesized in some studies (Pscheidt et al., 2019). Mehlenbacher et al. (2007) reported a low frequency of fungi in Oregon and Barcelona cultivars, while Turkish cultivars showed higher fungi levels (Ozay et al., 2008). In our study, we found that hazelnuts from both Oregon and Turkey cultivars exhibited low levels of fungus. Therefore, despite the aforementioned results, it is foolhardy to believe that the differences in contamination levels depend exclusively on the cultivar or the origin. As reported by several studies, fungi represent the main microbial contaminants in raw hazelnuts, and their presence is affected by numerous factors such as the cultivation area climate, harvest time, and storage condition (Kader, 2013; Battilani et al., 2018; Pscheidt et al., 2019). Fungal contamination is reduced by drying but not completely removed. In fact, among many fungi belonging to the order Eurotiales, the environmentally ubiquitous genera *Aspergillus* and *Penicillium* can survive high temperatures and can exhibit other types of extreme stress resistance (Dijksterhuis, 2007; Wyatt et al., 2015). The persistence of specific fungi in the drying process was also highlighted by Valente et al. (2020), who found that *A. flavus*, intentionally inoculated at a final concentration of 1^7^ spores/ml in hazelnuts, reached levels of about 6.1^6^ CFU/g when dried at 35°C. Based on these results and consideration, the drying process, while ensuring chemical–physical characteristics compatible with ready-to-eat products, does not remove the fungal danger and the consequent mycotoxins risk. To date, alternative strategies, such as UV or gamma rays irradiation, ultrasound, atmospheric and low-pressure plasma technology, as well as chemical treatments based on the use of hydrogen peroxide, ethylene, acids, or bases, have been developed for the prevention of toxigenic fungal in hazelnuts (Di Stefano et al., 2014; Mao et al., 2016; Şen, 2021). However, these methods are often impractical because they are too expensive and difficult to implement. or time consuming. For this purpose, other approaches including biological control using antagonistic microorganisms could be more effective and consistent with environmental and human health. Therefore, to identify a bioprotective strategy for hazelnuts subjected to drying, the isolation and species-level identification of various post-harvest fungi resistant to the drying process is a key step.

### Identification of Fungi in Dry Hazelnuts

#### Microscopic Features of Fungi

**FIGURE 3:**
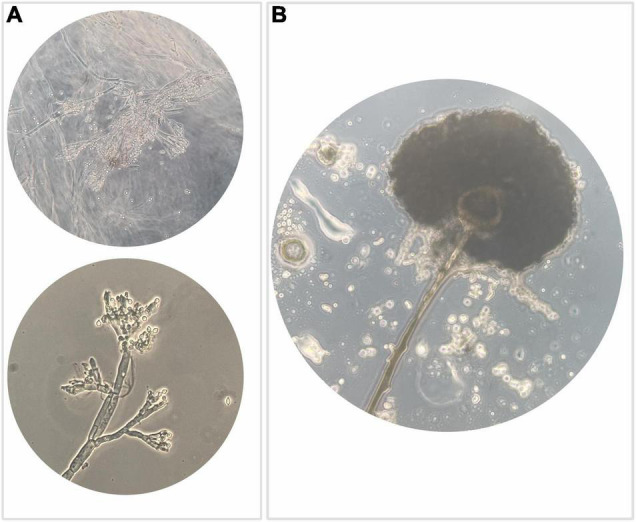
Optical microscope observation highlighting: **(A)** typical structure of agamic reproduction of the genus *Penicillium*, which is shaped like a “brush” with a basal conidiophore branch from which the *metulae* differentiate, inserted rod-like structures that in turn branch out with phialides that generate a chain of conidia usually roundish; **(B)** anamorphic reproductive structures of the genus *Aspergillus*, which include a conidiophore branch that at the apex differentiates a spheroidal vesicle that generates *metulae* that in turn give rise to phialides responsible to produce *conidia*.

Isolates (*N* = 350) were examined for cultural, microscopic, and morphological characteristics. Most of the isolates (*N* = 134) highlighted the typical structure of agamic reproduction of the *Penicillium* genus (Figure 3A) and 68 isolates showed characteristics compatible with the *Aspergillus* genus (Figure 3B). The predominant presence of fungi attributable to *Penicillium* and *Aspergillus* genera is not only due to the fact that the two genera are usually found in raw hazelnuts but also to their recognized resistance to ecological factors.

Many fungi belonging to *Aspergillus* and *Penicillium* are ubiquitous and form sexual ascospores that can survive at high temperatures and can exhibit other types of extreme stress resistance (Dijksterhuis, 2007; Wyatt et al., 2015; Dasan et al., 2016). Remarkable is also the occurrence of isolates (*N* = 54) with characteristics compatible with *Fusarium*.

**FIGURE 4:**
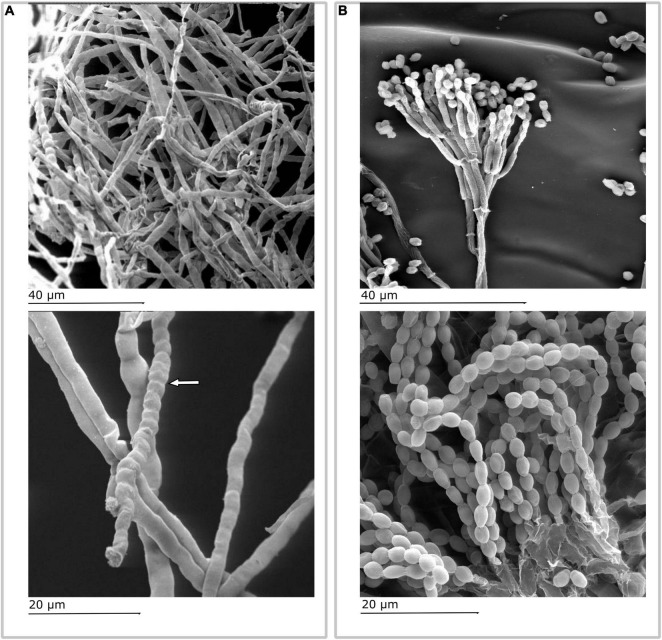
Scanning electron microscopy images: **(A)** *Fusarium* spp. mycelium. In highlight (white arrow), the presence of chlamydospores characterized by a thickened wall and rich in reserve substances capable of surviving in adverse environmental conditions and remain quiescent for long periods waiting for favorable conditions; **(B)** *Penicillium* spp.: typical branched conidiophore with chains of conidia.

Literature reported (Rodrigues et al., 2012) that the genus *Fusarium* is predominant in the field and in postharvest. Based on our observations, we could assume that the presence of isolates referable to this genus can be related to their ability to resist adverse environmental conditions. In this regard, the evidence found by SEM observation is of interest (Figure 4A) that highlighted the presence of chlamydospores characterized by a thickened wall and rich in reserve substances capable of surviving in adverse environmental conditions. Figure 4B, obtained from SEM observation, shows a typical branched conidiophore with chains of conidia in *Penicillium* spp.

The remaining isolates were distinguished by characteristics compatible with the other three genera *Rhizopus* (*N* = 38), *Alternaria* (*N* = 38), and *Trichoderma* (*N* = 18).

Other authors (Jiménez et al., 1991; Hong et al., 2006; Arciuolo et al., 2020; Wei et al., 2020) have also found a high incidence not only of *Aspergillus* and *Penicillium* genera but also of *Fusarium* and *Alternaria* genera in various samples of hazelnuts.

In our study, unlike the findings of Arciuolo et al. (2020) (although conducted over a wider geographic range), in several batches from the Caucasian range, no isolates possessing characteristics compatible with *Diaporthe* were found.

#### Inter and Intra Genera Diversity

The results of microscopic and morphological examination suggest reasonable biodiversity in fungal contamination. Keeping in mind that biodiversity is a major contributor to the occurrence of hazards in post-harvest produce, species-level contaminant identification is the key step in developing an important and efficient plan to reduce fungal growth and protect food products (Tremonte et al., 2017a; Sorrentino et al., 2018; El Sheikha, 2019). It is known that protective and antimicrobial tools (microbial and natural compounds) are often species-specific (Sorrentino et al., 2013; Tremonte et al., 2016; Iorizzo et al., 2020b).

**FIGURE 5:**
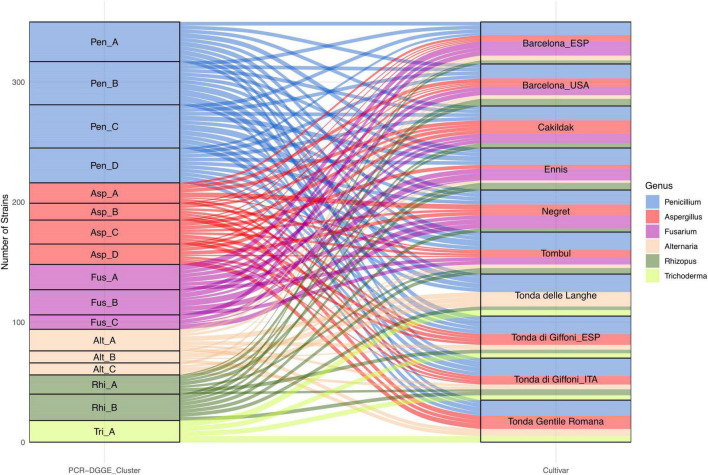
Alluvial plot showing the frequency of fungi genus grouped in six PCR-DGGE clusters and isolated from dried hazelnuts belonging to eight cultivars cultivated and processed in 10 geographical areas.

In our study, the species level was detected through the PCR-DGGE analysis, which has already been described as an effective and convenient means of profiling not only the bacterial (Tremonte et al., 2017b) but also the fungal community (El Sheikha, 2019). Based on the variability of sequences within rDNA regions among different microbial species, each species is assumed to produce a different PCR-DGGE profile. Considering a similarity level of 80% as the arbitrary threshold for the identification at the species level, the fungi isolates were grouped into six clusters and 17 subclusters (Figure 5). Cluster analysis, grouping the totality of isolates into six primary groups, confirms the results already found through microscopic and morphological analysis.

*Penicillium* and *Aspergillus* remain the predominant genera with 134 and 68 isolates, respectively. Moreover, certain interspecies biodiversity emerges within the two genera. The 134 isolates referable to the genus *Penicillium* have been grouped into four clusters, each potentially referable to a different species.

In addition, other four clusters have been identified within the isolates referable to the genus *Aspergillus*. Both genera showed a wide distribution. In particular, *Penicillium* isolates, regardless of the PCR-DGGE cluster, were detected in all assayed batches. Isolates from the four clusters of *Aspergillus* were found in all batches except for the Tonde delle Langhe batches. The 54 fungi characterized, and compatible with the *Fusarium* genus showed PCR-DGGE profiles grouped in three different clusters suggesting that they belong to three different taxonomic species. Interestingly, the results highlight that *Fusarium* spp. seem to show some specificity for cultivar and not for the geographical area. *Fusarium* isolates were found in Cakildak, Ennis, Negret, and Tombul batches, as well as in Barcelona from both Oregon and Catalonia; while resulting undetectable in Tonda delle Langhe, Tonda Romana, and Tonda di Giffoni regardless of the geographical area (Catalonia or Southern Italy). Several authors have already reported that geographical area and cultivar could affect microbial susceptibility (Kabak, 2016; Houshyarfard and Javadi, 2018; Silvestri et al., 2021). However, there is not much information regarding the relationship between individual fungal species and the different cultivars most prevalent in the northern hemisphere.

The occurrence of *Trichoderma* spp. also suggests a specific susceptibility of certain cultivars. Specifically, isolates referable to *Trichoderma*, all characterized by the same PCR-DGGE profile, were only found in samples from Italian cultivars. On the contrary, *Alternaria* and *Rhizopus* isolates showing three and two different PCR-DGGE profiles, respectively, were found in almost all batches, except Negret batches for *Alternaria* spp. and Tonda Gentile Romana ones for *Rhizopus*. Based on the findings described earlier, our study, for the first time, highlighted the biodiversity in hazelnut fungi genera and emphasized the relationship between PCR-DGGE profile and specific cultivars.

#### Species Diversity

**FIGURE 6:**
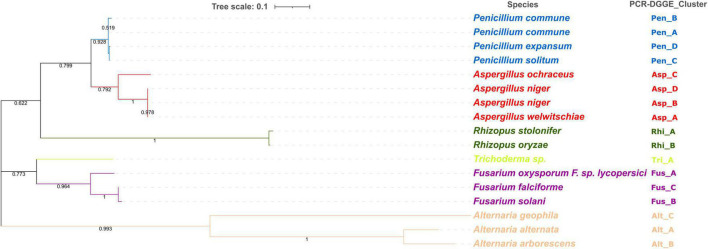
Phylogenetic analysis of ITS region of 17 isolates (*Penicillium* spp., *N* = 4; *Aspergillus* spp., *N* = 4; *Rhizopus* spp., *N* = 2; *Trichoderma* spp., *N* = 1; *Fusarium* spp., *N* = 3; *Alternaria* spp., *N* = 3). Phylogenetic tree calculation was performed with the Neighbor-Joining method and using MEGAX software. Statistical significance has been tested by using bootstrap analysis.

The PCR-DGGE enables not only the study of microbial diversity but also can be coupled with other techniques such as cloning and subsequent sequencing to analyze the specific DNA sequences (Theron and Cloete, 2000). According to the migration profiles, for each PCR-DGGE-gel, one strain from each subcluster was selected for subsequent genetic sequencing. A total of 17 isolates were sequenced and the results (Figure 6) allowed for identification at the species level. Other authors have already highlighted the importance of the choice of amplicons in the description of fungal communities (De Filippis et al., 2017; Sha et al., 2017). The results obtained from sequence analyses of the internal transcribed spacer (ITS) region ITS1—5.8S—ITS2 rDNA allowed us to identify 15 different species. Combining these results with those obtained from the PCR-DGGE profiles cluster analysis, it was possible to identify at species levels all the isolates. Specifically, the PCR-DGGE profile of *Penicillium* isolates corresponded to three different species: 69 isolates, grouped in PCR-DGGE clusters Pen_A and Pen_B, were identified as *Penicillium commune* (*P. commune*); 36 isolates were identified as *Penicillium solitum* (*P. solitum*); and 29 isolates as *Penicillium expansum* (*P. expansum*).

Based on our findings highlighting the prevalence of the three species in all hazelnut lots and considering the available knowledge regarding their relationship to consumer health, a concerning situation can be perceived. *P. commune* is recognized as a producer of cyclopiazonic acid (CPA), a mycotoxin often detected in foods of plant origin nut (Fernandez Pinto et al., 2001; Prencipe et al., 2018). Not to be underestimated is also the presence of *P. expansum* and *P. solitum*. Strains belonging to *P. expansum* were often isolated from other types of nuts (Donis-González et al., 2016; Spadaro et al., 2021) and could produce patulin, chaetoglobosin A, and roquefortine (Donis-González et al., 2016; Spadaro et al., 2021). Moreover, both *P. expansum* and *P. solitum* are reported as pathogens on chestnuts and are able to adapt to different environments (Louw and Korsten, 2014; Donis-González et al., 2016; Scholtz and Korsten, 2016). Three different species were, also, detected within PCR-DGGE clusters related to *Aspergillus* isolates.

In detail, 31 isolates were identified as *Aspergillus nige*r, 20 as *Aspergillus ochraceus*, and 17 as *Aspergillus welwitschiae.*

*Aspergillus* is recognized as the most common genera causing nuts spoilage, and their ingestion may cause mycoses, especially in immunocompromised patients (Yassein et al., 2021). In fact, despite the industrial importance of both *Aspergillus nige*r and *Aspergillus ochraceus*, they are recognized as producers of mycotoxins, namely ochratoxin A (OTA) and fumonisin B2 (FB2) (Frisvad et al., 2004, 2007; Dasan et al., 2016). In addition, the other species found, such as *A. ochraceus*, are described as potential ochratoxin-producing species (Ghitakou et al., 2006).

The two *Rhizopus* PCR-DGGE clusters found in all batches, except for Tonda Gentile Romana, were identified as *R. stolonifer* and *R. oryzae.* Other authors have already described these two species as common contaminants of freshly harvested nuts and being persistent during storage (Pitt and Hocking, 2009).

The identification at genus levels of isolates from the *Trichoderma* PCR-DGGE cluster was confirmed by sequencing results without actually describing the species level.

Conversely, identification at the species level was achieved for isolates from three *Alternaria* clusters and three *Fusarium* clusters.

Species of the *Fusarium* and *Alternaria* genus are readily found in the air transported as spores or cells on particles in aerosols from plants, animals, and soil, and their origin can be food (from fruits, leaves, and vegetables), water (in fresh or contaminated drainage water), and soil (soil particles on food or in the air), and their concentration varies by area, season, and climate (Gisi, 2022). They have been reported as plant pathogens in several studies. In particular, species of *Fusarium* are associated with citrus dry rot in California and Texas (Adesemoye and Eskalen, 2011; Kunta et al., 2015), crown rot, and stem canker of pistachio rootstocks in central California (Crespo et al., 2019). However, they are a very rare serious human pathogen, although *Fusarium solani* (>60%) and the species complex *F. oxysporum* (<20%) are often associated with human fusariosis (Al-Hatmi et al., 2016; Al-Maqtoofi and Thornton, 2016; Kischkel et al., 2020).

In our study, out of 54 isolates belonging to *Fusarium* spp., 21 isolates were identified as *Fusarium oxyxporum*, 21 as *Fusarium solani*, and 12 as *Fusarium falciforme*. Three different species have been also detected among *Alternaria* showing *A. alternata* as the most recurrent, followed by *A. geophila* and *A. arborescens*. Alternaria species are notable for the production of several compounds, such as alternariol monomethyl ether and tenuazonic acid, that are notable for their toxicity and disease-causing effects on humans (Gotthardt et al., 2020; Oyedeji et al., 2021).

## Conclusion

The results implement the knowledge available in the literature on hazelnut treatments by highlighting that the hazelnut drying process does not eliminate microbiological and therefore mycotoxigenic risks. Regardless of the geographical area of production, dried hazelnut samples harbor a significant and diverse fungal community. In the assayed samples, fungi from six different genera were isolated, often showing certain biodiversity at the species level. *Penicillium* and *Aspergillus* species were found in all varieties of dried hazelnuts from different geographical areas. A “hazelnut variety-fungal genus” specificity seems to characterize some genera such as *Fusarium* and *Alternaria*. The integration of the data obtained with the information available in the literature shows that the fungal species that have been found constitute a potential health hazard for the consumer. The species-level description of the most commonly occurring fungi in dried hazelnuts is an important starting point for the subsequent identification of protective biotechnologies.

## Data Availability Statement

The original contributions presented in the study are included in the article/supplementary material, further inquiries can be directed to the corresponding author.

## Author Contributions

SL: involved in experimental designing and fungi genotypic characterization. GP: data interpretation and figure elaboration, drafting, and revising the study. PT: design of the study, analysis and interpretation of data, and drafting and revising the study. IM: fungi isolation and identification. FV: involved in genotypic characterization. CC: sampling and batches selection. LM: fungi microscopic features description. MI: drafting and revising the study. MS: revising the study. ES: involved in experimental designing and drafting of the study. RC: agreement to be accountable for all aspects of the study in ensuring that questions related to the accuracy or integrity of any part of the study were appropriately investigated and resolved. All authors contributed to the article and approved the submitted version.

## Conflict of Interest

The authors declare that the research was conducted in the absence of any commercial or financial relationships that could be construed as a potential conflict of interest.

## Publisher’s Note

All claims expressed in this article are solely those of the authors and do not necessarily represent those of their affiliated organizations, or those of the publisher, the editors and the reviewers. Any product that may be evaluated in this article, or claim that may be made by its manufacturer, is not guaranteed or endorsed by the publisher.
